# Employment of a Resazurin Viability-Based Assay for Minimum Inhibitory and Bactericidal Concentration Determination

**DOI:** 10.3390/ph19030505

**Published:** 2026-03-19

**Authors:** Lorena G. Calvo, Stephanya Corral-Orbe, Rosa-Antía Villarino, Sandra Sánchez, Trinidad de Miguel

**Affiliations:** 1 Department of Microbiology and Parasitology, Universidade de Santiago de Compostela, 15782 Santiago de Compostela, Spain; lorena.gomez.calvo@rai.usc.es (L.G.C.); mishellstephanya.corral@usc.es (S.C.-O.); rosaantia.villarino@rai.usc.es (R.-A.V.); sandra.sanchez@usc.es (S.S.); 2 Galicia Sur Health Research Institute (IIS Galicia Sur), SERGAS-UVIGO, 36213 Vigo, Spain; 3 Agribiotechnology and Precision Breeding for Food Security National Laboratory, Institute of Genetics and Biotechnology, Hungarian University of Agriculture and Life Sciences (MATE), 2100 Gödöllő, Hungary

**Keywords:** resazurin, microbiological methods, MBC, MIC, EUCAST, antibiotic resistance, foodborne

## Abstract

**Background/Objectives**: The increasing prevalence of antimicrobial-resistant bacteria highlights the need for improved methodologies to evaluate antimicrobial activity beyond conventional minimum inhibitory concentration testing. While resazurin-based assays are widely used for minimum inhibitory concentration determination due to their simplicity and sensitivity, minimum bactericidal concentration assessment still relies on labor-intensive colony-forming unit counting. The objective of this study was to develop and validate a resazurin-based microwell assay capable of determining both the minimum inhibitory concentration and the minimum bactericidal concentration without routine plate counting, thereby simplifying bactericidal evaluation. **Methods**: A two-step resazurin-based fluorescence assay was designed and performed in microplates. After determining the minimum inhibitory concentration using resazurin as a metabolic indicator, well-showing inhibited bacterial growths were subjected to a regrowth phase by transferring aliquots into fresh antimicrobial-free medium containing resazurin. This additional step allowed discrimination between reversible metabolic inhibition and irreversible bacterial death. The method was evaluated using ciprofloxacin and chloramphenicol against four bacterial species: *Staphylococcus aureus*, *Enterococcus faecalis*, *Escherichia coli*, and *Pseudomonas aeruginosa*. Minimum bactericidal concentration values obtained using this assay were compared with those obtained through conventional colony counting on agar plates. **Results**: Minimum bactericidal concentration values obtained using the two-step fluorescence assay were fully concordant with the conventional colony-forming unit counting method for all tested antibiotics and bacterial species. **Conclusions**: The proposed two-step resazurin-based microwell assay represents a rapid, reliable, and less labor-intensive alternative for the determination of both the minimum inhibitory concentration and the minimum bactericidal concentration, with potential applications in clinical and industrial microbiology laboratories.

## 1. Introduction

Resazurin is a blue, non-fluorescent dye that is reduced to the pink, fluorescent resorufin by metabolically active bacterial cells [1]. This redox-based colorimetric assay provides a simple, rapid, and reliable method for assessing bacterial viability and is commonly employed in determining antimicrobial efficacy [2]. Because this conversion depends on cellular metabolic activity, resazurin-based assays have become widely adopted for rapid and sensitive determination of minimum inhibitory concentrations (MICs). However, MIC determination reflects only growth inhibition and does not provide information about bactericidal activity. The minimum bactericidal concentration (MBC), defined as the lowest concentration of an antimicrobial agent that results in complete loss of viable bacteria, remains essential for distinguishing bacteriostatic from bactericidal compounds [3]. This distinction is particularly relevant in non-clinical and abiotic contexts, including food processing, surface sanitation, and medical device disinfection, where complete eradication of microorganisms is required [4].

Despite its importance, MBC determination is less utilized than MIC testing, as current antimicrobial susceptibility guidelines, such as those from the European Committee on Antimicrobial Susceptibility Testing (EUCAST) and Clinical and Laboratory Standards Institute (CLSI), primarily focus on inhibitory endpoints, without addressing complete viability eradication [3,5,6]. Traditional MBC relies on CFU enumeration through agar plating, a time-consuming, labor-intensive, and resource-demanding procedure that limits its applicability in antimicrobials screening [7]. While resazurin assays are extensively employed for MIC evaluation, their application to MBC determination remains underexplored [2,8,9,10,11,12]. Because resazurin reduction reflects metabolic activity rather than definitive cell death, a single-step assay cannot distinguish between temporary metabolic suppression and irreversible bacterial killing. Therefore, methodological adaptation is required to extend its use beyond inhibitory assessment.

To address this limitation, we validated a two-step resazurin-based assay incorporating a regrowth phase in fresh antimicrobial-free medium [13]. This approach is based on the hypothesis that bacteria inhibited by sublethal concentrations of antimicrobials are capable of regrowing in fresh supportive media, with this regrowth being detected using resazurin, as already established for MIC assays [12]. In this context, MIC and MBC values may coincide for some antimicrobials if the tested agent acts as a bactericidal. However, true MBC determination requires a re-culture step to confirm the complete loss of bacterial viability, something not possible when performing conventional resazurin assays alone [12]. Hence, incorporating a resazurin-based regrowth step may be an effective tool for a rapid and wide-concentration antimicrobial screening alternatively to traditional plate counting.

In the present study, we evaluated this optimized methodology using ciprofloxacin and chloramphenicol against representative Gram-positive and Gram-negative bacteria, including *Staphylococcus aureus*, *Enterococcus faecalis*, *Escherichia coli*, and *Pseudomonas aeruginosa*, well-known surface contaminants and nosocomial pathogens, classified as highly virulent and antibiotic-resistant ESKAPE bacterial pathogens [14]. By comparing fluorescence-based MBC values with conventional CFU enumeration, we demonstrate that the proposed method provides a reliable, rapid, and cost-effective alternative for simultaneous MIC and MBC determination. This strategy may facilitate antimicrobial screening in both clinical and industrial microbiology laboratories.

## 2. Results

The performance of the two-step resazurin-based method was evaluated by comparing its MBC results with those obtained using the conventional colony-forming unit plating method, the gold-standard methodology for MBC determination.

Following the initial MIC determination, aliquots from wells showing no visible growth were transferred to fresh medium and incubated to assess bacterial regrowth.

**Figure 1:**
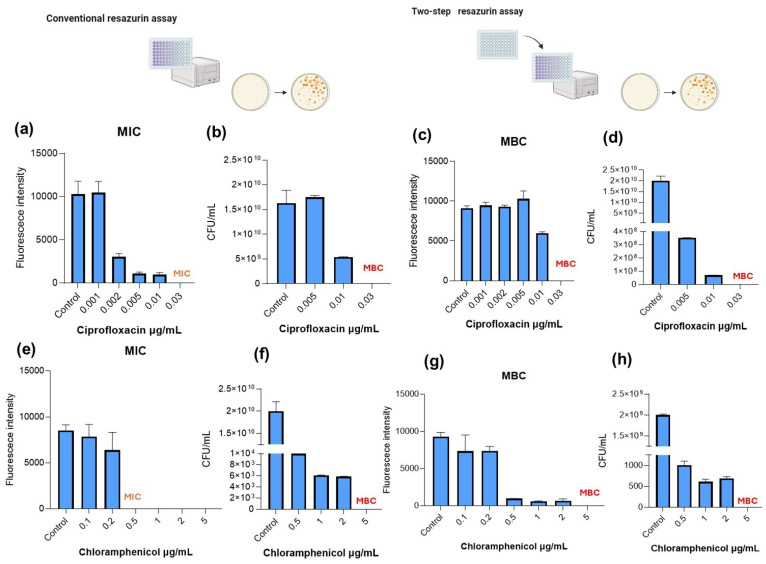
Determination of the minimum bactericidal concentration (MBC) of ciprofloxacin and chloramphenicol against *S. aureus* using conventional plate counting and the two-step resazurin fluorescence assay. (**a**) Ciprofloxacin MIC determined by the conventional resazurin assay. (**b**) MBC determined by plate counting following the conventional resazurin assay. (**c**) MBC determination using fluorescence measurements in the two-step resazurin assay. (**d**) MBC validation by plate counting after the two-step resazurin assay. (**e**) Chloramphenicol MIC determined by the conventional resazurin assay. (**f**) MBC determined by plate counting following the conventional resazurin assay. (**g**) MBC determination using fluorescence measurements in the two-step resazurin assay. (**h**) MBC validation by plate counting after the two-step resazurin assay. Scheme figures were created with Biorender.com.

**Figure 2:**
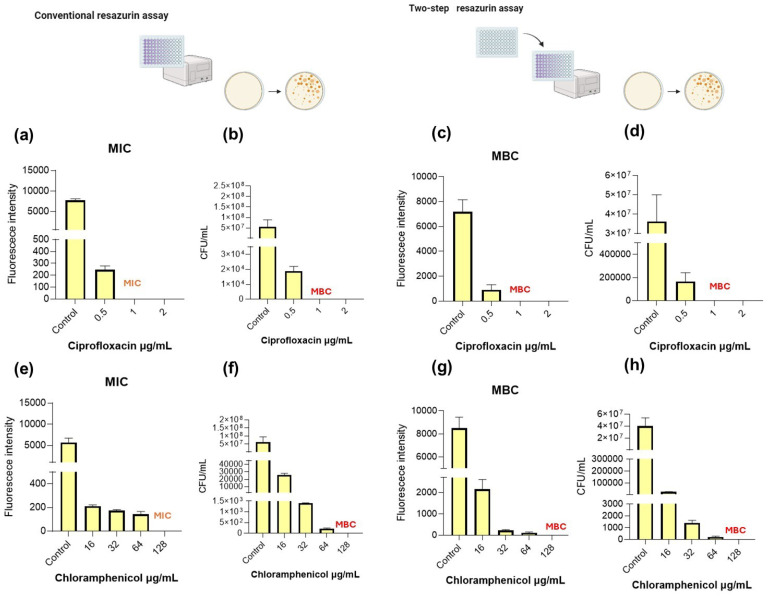
Determination of the minimum bactericidal concentration (MBC) of ciprofloxacin and chloramphenicol against *E. faecalis* using conventional plate counting and the two-step resazurin fluorescence assay. (**a**) Ciprofloxacin MIC determined by the conventional resazurin assay. (**b**) MBC determined by plate counting following the conventional resazurin assay. (**c**) MBC determination using fluorescence measurements in the two-step resazurin assay. (**d**) MBC validation by plate counting after the two-step resazurin assay. (**e**) Chloramphenicol MIC determined by the conventional resazurin assay. (**f**) MBC determined by plate counting following the conventional resazurin assay. (**g**) MBC determination using fluorescence measurements in the two-step resazurin assay. (**h**) MBC validation by plate counting after the two-step resazurin assay. Scheme figures were created with Biorender.com.

**Figure 3:**
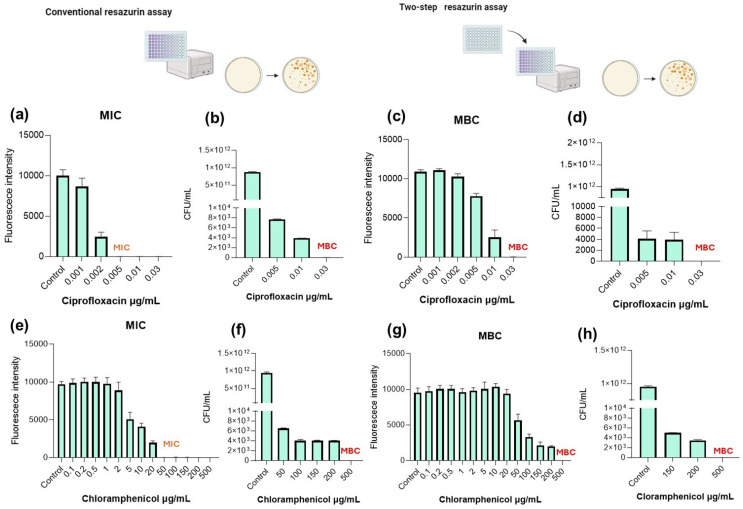
Determination of the minimum bactericidal concentration (MBC) of ciprofloxacin and chloramphenicol against *P. aeruginosa* using conventional plate counting and the two-step resazurin fluorescence assay. (**a**) Ciprofloxacin MIC determined by the conventional resazurin assay. (**b**) MBC determined by plate counting following the conventional resazurin assay. (**c**) MBC determination using fluorescence measurements in the two-step resazurin assay. (**d**) MBC validation by plate counting after the two-step resazurin assay. (**e**) Chloramphenicol MIC determined by the conventional resazurin assay. (**f**) MBC determined by plate counting following the conventional resazurin assay. (**g**) MBC determination using fluorescence measurements in the two-step resazurin assay. (**h**) MBC validation by plate counting after the two-step resazurin assay. Scheme figures were created with Biorender.com.

**Figure 4:**
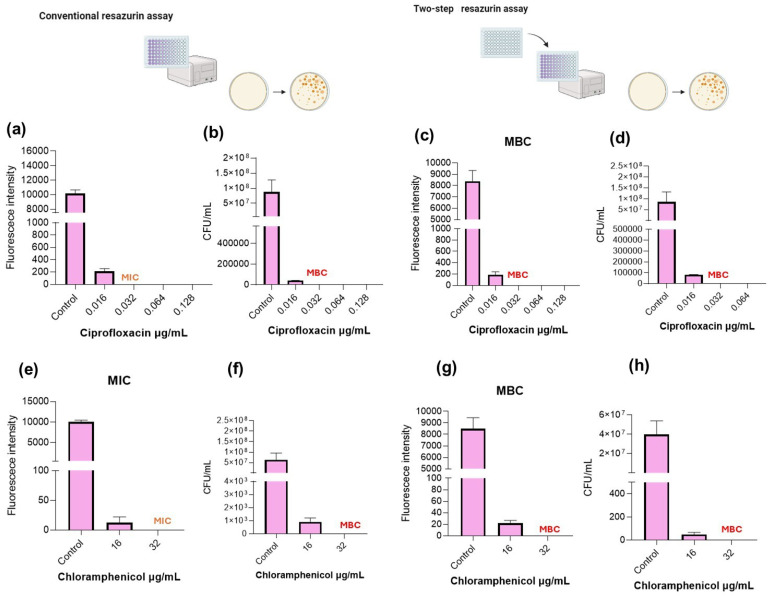
Determination of the minimum bactericidal concentration (MBC) of ciprofloxacin and chloramphenicol against *E. coli* using conventional plate counting and the two-step resazurin fluorescence assay. (**a**) Ciprofloxacin MIC determined by the conventional resazurin assay. (**b**) MBC determined by plate counting following the conventional resazurin assay. (**c**) MBC determination using fluorescence measurements in the two-step resazurin assay. (**d**) MBC validation by plate counting after the two-step resazurin assay. (**e**) Chloramphenicol MIC determined by the conventional resazurin assay. (**f**) MBC determined by plate counting following the conventional resazurin assay. (**g**) MBC determination using fluorescence measurements in the two-step resazurin assay. (**h**) MBC validation by plate counting after the two-step resazurin assay. Scheme figures were created with Biorender.com.

Figure 1, Figure 2, Figure 3 and Figure 4 show the MIC and MBC determination of ciprofloxacin and chloramphenicol of *S. aureus* and *E. faecalis* as Gram-positive bacteria and *E. coli* and *P. aeruginosa* as representative of Gram-negative bacteria.

### 2.1. S. aureus

The antimicrobial activity of ciprofloxacin and chloramphenicol against *S. aureus* was evaluated using both the conventional resazurin assay (MIC determination) and the proposed two-step resazurin method (MBC determination), with confirmation by CFU counting. In the conventional resazurin assay (Figure 1a), fluorescence intensity remained high at 0.001 µg/mL, indicating metabolic activity. A sharp decrease in fluorescence was observed at 0.002–0.005 µg/mL, with almost complete loss of signal at 0.01 µg/mL, establishing the MIC at 0.03 µg/mL where no signal was detected. CFU enumeration (Figure 1b) corroborated these findings, showing a marked reduction in viable counts at 0.01 µg/mL. However, detectable CFUs were still present, indicating growth inhibition but not complete killing. Following the regrowth step performed in the resazurin two-step assay (Figure 1c), fluorescence remained high at concentrations below 0.03 µg/mL, demonstrating bacterial recovery in fresh medium and that only at 0.03 µg/mL the fluorescence was abolished, indicating absence of viable cells.

CFU analysis confirmed complete bacteria eradication at 0.03 µg/mL, establishing the MBC at 0.03 µg/mL (Figure 1d).

Therefore, for ciprofloxacin, MIC and MBC values coincided for *S. aureus* (0.03 µg/mL). Importantly, the two-step resazurin assay accurately identified the bactericidal concentration, matching CFU results.

Regarding chloramphenicol, in the conventional resazurin assay (Figure 1e), fluorescence progressively decreased with increasing concentrations and the MIC was determined at 0.5 µg/mL. CFU data showed cell reduction at 0.5 µg/mL; however, viable bacteria were detected (Figure 1f). The two-step resazurin assay was employed for MBC determination as well as CFU counting (Figure 1g,h). After the regrowth step, fluorescence persisted at 0.5 and 1 µg/mL, indicating that bacteria were able to recover and MBC was set at 5 µg/mL. CFU results, shown in Figure 1h, confirmed that complete bacterial eradication occurred only at 5 µg/mL, establishing the MBC at 5 µg/mL.

Contrary to ciprofloxacin, the MBC for chloramphenicol (5 µg/mL) was substantially higher than the MIC (0.5 µg/mL), indicating a predominantly bacteriostatic effect against *S. aureus.*

### 2.2. E. faecalis

*E. faecalis* is a more resistant bacteria in comparison to *S. aureus,* which has shown to be quite sensitive to the tested antibiotics. Regarding *E. faecalis*, in the conventional resazurin assay (Figure 2a) fluorescence intensity was markedly reduced at 0.5 µg/mL, with complete suppression observed at 1 µg/mL, with this concentration being set as MIC. CFU quantification showed a substantial reduction in viable counts at 0.5 µg/mL, although detectable colonies remained, as shown in Figure 2b, at 1 µg/mL; CFUs were drastically reduced, with this concentration being considered the MBC.

In order to validate the two-step resazurin assay as a reliable method for MBC determination, its results were compared with the plate count method. As observed in Figure 2c, fluorescence remained detectable at 0.5 µg/mL, indicating bacterial growth; in contrast, no fluorescence was detected at 1 µg/mL and 2 µg/mL, suggesting complete loss of viability. CFU enumeration (Figure 2d) confirmed the absence of viable bacteria at 1 µg/mL, establishing the MBC at 1 µg/mL, with this coinciding with the result provided by the two-step resazurin assay.

The same tendency was observed when testing chloramphenicol. In the conventional resazurin assay (Figure 2e), fluorescence intensity decreased progressively with increasing chloramphenicol concentrations, with the MIC being determined at 128 µg/mL, based on complete inhibition of metabolic activity. CFU results (Figure 2f) supported these findings, showing a strong reduction in bacterial counts at high concentrations, although cell eradication, MBC, was determined at 128 µg/mL.

The two-step resazurin assay and CFU counts prove that complete bacterial elimination was reached at 128 µg/mL, establishing this concentration as the MBC.

### 2.3. P. aeruginosa

*P. aeruginosa* is a Gram-negative bacterium well known for its antibiotic resistance. When exposed to ciprofloxacin, in the conventional resazurin assay, fluorescence intensity remained high at 0.001 µg/mL, indicating metabolic activity. A marked reduction in fluorescence was observed at 0.002 µg/mL, with complete suppression at 0.005 µg/mL and above. The MIC was therefore determined at 0.005 µg/mL. CFU enumeration (Figure 3b) showed a substantial reduction in viable counts at 0.005 µg/mL, although residual CFUs were still detected, indicating growth inhibition rather than complete killing. For MBC determination, two-step assay (Figure 3c) and CFU counting (Figure 3d) were performed, demonstrating bacterial recovery in fresh medium and only complete bacterial eradication at 0.03 µg/mL. This test showed completely different MIC and MBC values for ciprofloxacin when *P. aerugonisa* was tested and proved that the two-step resazurin assay accurately identified the bactericidal concentration in agreement with CFU plating.

When tests were performed using chloramphenicol as antimicrobial, MIC was determined at 50 µg/mL, where fluorescence was substantially reduced, as shown in Figure 3e. However, CFU analysis (Figure 3f) showed a progressive reduction in viable counts with increasing concentrations, but detectable viability remained until 500 µg/mL.

After the regrowth step (Figure 3g), fluorescence persisted at concentrations up to 150–200 µg/mL, indicating bacterial recovery, and only at 500 µg/mL fluorescence was eliminated. CFU enumeration (Figure 3h) confirmed complete eradication only at 500 µg/mL, establishing this concentration as the MBC, being coincident with the result proposed by the resazurin assay.

### 2.4. E. coli

In the conventional resazurin assay (Figure 4a), fluorescence intensity was reduced at 0.016 µg/mL, with complete suppression observed at 0.032 µg/mL and above. Based on metabolic inhibition, the MIC was established at 0.032 µg/mL. CFU enumeration (Figure 4b) showed a strong reduction in viable counts at 0.016 µg/mL, although residual colonies were still detected. At 0.032 µg/mL, CFUs were nearly abolished, supporting the MIC determination.

For MBC determination, following the regrowth step (Figure 4c), fluorescence persisted at 0.016 µg/mL, indicating bacterial recovery in fresh medium. In contrast, fluorescence was abolished at 0.032 µg/mL and higher concentrations. Figure 4d confirmed complete absence of viable bacteria at 0.032 µg/mL, establishing the MBC at this concentration. For ciprofloxacin, MIC and MBC coincided at 0.032 µg/mL, indicating a clear bactericidal effect against *E. coli.* The two-step resazurin assay accurately identified the bactericidal concentration, fully concordant with CFU plating results. The same tendency was determined when testing chloramphenicol (Figure 4e–h). MIC determination using the conventional resazurin assay established 32 µg/mL as the inhibitory value. CFU analysis demonstrated no growth at 32 µg/mL, coinciding with the two-step resazurin assay recovery test, which identified this same concentration as MBC, as shown in Figure 4g.

In general, the MBC values obtained using the resazurin regrowth step were consistent with those determined by CFU counting for the tested bacteria and antimicrobials.

## 3. Discussion

Identification of new antimicrobials has become an evolving research area due to the urgent need to combat antibiotic-resistant bacteria, especially in nosocomial environments [15]. This increasing prevalence of antimicrobial resistance has intensified the need for rapid, reliable, and scalable methods for antimicrobial screening. While MIC determination remains the gold standard for susceptibility testing in clinical practice, it reflects only growth inhibition [16]. However, bactericidal activity becomes particularly relevant in non-clinical environments such as food processing, surface disinfection, and medical device sterilization, where complete pathogen eradication is required [4,17].

Additionally, sublethal exposure to antimicrobials has been associated with adaptive responses including enhanced biofilm formation, oxidative stress tolerance, and alterations in membrane proteins, potentially contributing to cross-resistance [4,18]. Therefore, identifying true bactericidal concentrations is not only microbiologically relevant but also critical for preventing the emergence of resistance in environmental and industrial contexts.

Despite the importance of MBC determination, conventional plate counting remains the most employed method even though it is labor-intensive, time-consuming, and poorly suited for high-throughput screening [8,19]. Colorimetric and fluorometric methods have emerged as fast alternatives for antimicrobial screening, with resazurin being one of the most commonly reported in the literature [20,21,22,23,24,25]. Resazurin-based assays have long been used for MIC determination due to their sensitivity, simplicity, and compatibility with microplate formats. However, their application for bactericidal determination remains underexplored, since the conversion of resazurin to resorufin is dependent on bacterial metabolic reduction and then primarily indicates metabolic activity rather than irreversible cell death [26].

In this study, we demonstrate that incorporating a regrowth step into the conventional resazurin assay enables reliable MBC determination without the need for agar plating. The key conceptual advancement of this method lies in distinguishing temporary metabolic suppression from irreversible loss of viability. Bacteria exposed to sublethal concentrations may exhibit inhibited metabolic activity during initial incubation but maintain the ability to recover once transferred to fresh antimicrobial-free medium. The absence of resazurin reduction during this second incubation phase serves as an indicator of bactericidal activity.

For all the tested strains in this study, *S. aureus*, *E. faecalis*, *E. coli*, and *P. aeruginosa*, MBC values determined using the two-step resazurin method were in accordance with conventional CFU enumeration, and also clear differences between the conventional resazurin assay (MIC) and two-step resazurin assay (MBC) were determined in some of the strains and antibiotics. This validates the reliability of the fluorescence-based regrowth approach for MBC determination and demonstrates that simple methodological adaptations to the resazurin method can lead to different useful applications.

This methodological adaptation was previously described by our group for lactic acid bacteria growth determination, specifically to avoid resazurin deterioration caused by pH acidification [13]. During our evaluation of a plant extract, we also observed concordance between resazurin fluorescence and CFU counts when using the two-step assay, suggesting that this method is not limited to conventional antimicrobials but can also be applicable to complex natural compounds. Regarding the tested antibiotics, ciprofloxacin, a fluoroquinolone with well-established bactericidal activity, showed coincident or near-coincident MIC and MBC values in most strains, which is consistent with its mechanism of action targeting DNA gyrase and topoisomerase IV [27]. In contrast, chloramphenicol, classically described as bacteriostatic due to inhibition of protein synthesis [28], exhibited higher MBC than MIC values in several strains, particularly *S. aureus* and *P. aeruginosa*, confirming the ability of the assay to potentially discriminate between bacteriostatic and bactericidal profiles.

Before us, Kłodzińska et al. (2018) proposed a similar fluorescence-based methodology for MBC determination based on SYTO9 and propidium iodide live/dead cell specificity [29]. Although their method was rapid and sensitive, the high cost of the reagents may limit its application. In contrast, by distinguishing between temporary inhibition and true bactericidal activity, our method provides a rapid, scalable, and cost-effective alternative to conventional plate counting for bactericidal screening.

Batista et al. (2025) also proposed a regrowth-based method for determining minimum bactericidal concentration (MBC) using the SLOWMYCOI Sensititre™ assay for *Mycobacterium* species [12]. However, bacterial growth was assessed solely by optical determination, without validation through CFU counting. Furthermore, the use of resazurin has already demonstrated compatibility with colorimetric substances, including natural plant extracts [13,30,31,32,33] and other types of antimicrobials as organic and inorganic nanoparticles [9,33]. In previous work, Manso et al. (2023) applied this two-step resazurin approach to determine the MBC of a grape marc extract against nineteen antibiotic-resistant clinical bacterial isolates [31], showing the potential of this practical alternative to conventional CFU-based MBC determination, particularly when testing complex natural matrices.

Despite the clear improvements proposed by the described method and the correlation observed between the absence of fluorescence and CFU-based MBC determination, several methodological considerations must be considered in order to take maximum advantage of this method. (1) Differences in intrinsic metabolic rates between bacterial species, strains, or physiological states may influence fluorescence intensity independently of viable cell numbers, particularly in slow-growing organisms or metabolically quiescent subpopulations (2). Although the regrowth step substantially reduces colorimetric and turbidity-related interferences compared to single-step assays, complete elimination of such effects cannot be guaranteed in all experimental contexts, and therefore appropriate blanks for background subtraction should always be included. (3) The method provides a relative evaluation of bactericidal activity rather than absolute quantification of viable cells. While fluorescence recovery showed strong agreement with CFU plate counts within the tested conditions, absolute cell numbers cannot be directly inferred from fluorescence intensity alone; therefore, for applications requiring quantitative analysis, strain calibration curves correlating fluorescence signal with CFU/mL would be necessary. Nevertheless, for screening purposes, the ability to distinguish irreversible bacterial killing from mere growth inhibition is the primary objective of this method. In this context, the proposed assay offers a practical alternative to conventional plate counting for MBC determination.

## 4. Materials and Methods

### 4.1. Materials

Bacterial strains were purchased from the Spanish Type Culture Collection (CECT) (Valencia, Spain). *P. aeruginosa* ATCC 27853 and *E. coli* ATCC 25922 were selected as representatives of Gram-negative strains and *S. aureus* ATCC 25923 and *E. faecalis* CECT 795 were selected as Gram-positive strain examples.

The culture media TSA (Tryptone soy agar) was purchased from Condalab (Madrid, Spain) and the cation Adjusted Müller Hinton II broth (CAMHB) from Becton-Dickinson (BBL, Sparks, NV, USA). Chloramphenicol, ciprofloxacin and fetal bovine serum (FBS) were supplied by Sigma-Aldrich (St. Louis, MO, USA). The commercial resazurin solution alamarBlue from ThermoFisher Scientific (Waltham, MA, USA) was employed as enzymatic substrate for the resazurin cell viability test.

The commercial resazurin solution alamarBlue 10× concentrate was employed in this assay. If salt resazurin is employed, resazurin stock solution (100×) should be prepared by dissolving 0.50 g of resazurin sodium salt into 100 mL 1X PBS and subsequently 1:10 diluted in 1X PBS to prepare the resazurin working solution.

### 4.2. Bacterial Inoculum and Assay Plate Preparation

Bacteria were grown on TSA medium plates, which were incubated for 24 h at 37 °C. Plate preparation was performed as previously described by Calvo et al. (2025) [13]. In order to set the bacterial inoculum, a couple of colonies were transferred to 2 mL of CAMHB 2X and the optical density was measured at 600 nm wavelength and adjusted to match the 0.5 McFarland turbidity standard. The bacterial culture was then diluted in CAMHB 2X to a final inoculum size of 10^6^ colony-forming units per mL (CFU/mL). Following the EUCAST recommendations about fastidious bacteria medium supplementation, CAMHB was minimally supplemented with 4% FBS, to achieve the correct bacterial growth in *P. aeruginosa* assays. In brief, 100 μL of a bacterial culture was added to the wells of a 96-well microplate and mixed with 60 μL of phosphate-buffered saline (PBS) to control possible pH variations, as well as 40 μL of antimicrobial treatment in order to achieve a final well bacterial concentration of 5 × 10^5^ CFU/mL. Antibiotic stock solutions were prepared in water, ranging the tested concentrations from 0.001 to 0.128 µg/mL of ciprofloxacin and 0.1 to 500 µg/mL of chloramphenicol. Cell-free blanks were used in order to detect colorimetric fluorescence interferences.

### 4.3. Resazurin Assay for MIC Determination

The conventional fluorometric resazurin method was employed to determine the inhibitory effect of antibiotics. In addition, 96-well plates were prepared as described in Section 4.2 and incubated overnight at 37 °C. After the incubation period, 20 μL of alamarBlue resazurin solution was added to each well and re-incubated again until colorimetric change was observed (1–2 h for *S. aureus* and *E. coli* and 4–5 h for *P. aeruginosa* and *E. faecalis*). Finally, resazurin reduction to resorufin was measured using the FLUOstar microplate reader at an excitation wavelength of 544 nm and emission wavelength of 590 nm.

### 4.4. Resazurin Assay for MBC Determination

In order to determine the range of bactericidal concentration of antibiotics, a second resazurin plate incubation test was performed. This method was previously described by our group as an adapted methodology for lactic acid bacteria growth rate determination avoiding interferences between acidic metabolites and resazurin [13]. Briefly, the 96-well plates were prepared and incubated overnight as specified in Section 4.3. After the overnight incubation, 100 μL of fresh CAMHB 2X or CAMHB-FBS, 60 μL of PBS, 20 μL of resazurin solution and 20 μL of each well from the overnight incubated plate were mixed in a new 96-well microplate. As described previously, plates were incubated until colorimetric change was observed, conventionally 1–5 h or overnight in the case of *P. aeruginosa* and *E. faecalis*, which present slow-paced growth.

### 4.5. Plate Count Validation Assay

To test the accuracy of the fluorometric assays in MBC determination, bacterial strains were exposed to different extract concentrations as previously described in Section 4.3. Subsequently, 10^−0^,10^−1^, 10^−2^, 10^−4^, 10^−6^, 10^−8^ and 10^−10^ dilutions of the tested cultures were seeded on TSA. The plates were incubated at 37 °C overnight.

### 4.6. Statistical Analysis

All experiments were performed in triplicate. Fluorescence measurements were performed at an excitation wavelength of 544 nm and emission wavelength of 590 nm with a maximum fluorescence intensity of 10.000 under these conditions. Background interference from blank samples was subtracted from the fluorescence values. The medium and standard deviation of all samples were calculated and represented graphically using the software GraphPad Prism 9.0.

## 5. Conclusions

The development of reliable, rapid, and cost-effective methodologies for antimicrobial screening remains essential in the fight against antimicrobial resistance. The resazurin-based method proposed in this research addresses a critical gap in antimicrobial screening, by enabling MBC determination without the need for intensive plate counting. This method complements conventional MIC assessments enhancing the accuracy of susceptibility tests. By distinguishing temporary growth inhibition from irreversible bacterial death, this assay provides an effective tool for antimicrobial discovery and resistance monitoring. Its scalability, low cost, and adaptability to different bacterial strains and compounds make it a valuable tool for both clinical and industrial microbiology laboratories. As antimicrobial resistance continues challenging public health, adopting optimized fluorescence-based techniques such as this one may contribute to faster and more precise screening strategies, supporting better therapeutic decisions and safer antimicrobial applications.

## Data Availability

The original contributions presented in this study are included in the article. Further inquiries can be directed to the corresponding author.
